# A new method for integrated analysis applied to gene expression and cytokines secretion in response to LIPO-5 vaccine in HIV-negative volunteers

**DOI:** 10.1186/1742-4690-9-S2-P121

**Published:** 2012-09-13

**Authors:** R Thiebaut, B Liquet, H Hocini, S Hue, L Richert, M Raimbault, K Lê Cao, Y Levy

**Affiliations:** 1 INSERM U897, Bordeaux, France

## Background

We present an integrated analysis of gene expression and cytokine secretion using a novel statistical method applied to the ANRS VAC18 trial.

## Methods

The statistical approach combines multilevel and multivariate analyses. The statistical approach is based on a variance decomposition followed by a sparse Partial Least Square Discriminant Analysis (sPLS-DA) to select the discriminative features (genes, cytokines) separating the classes in a supervised framework, or sPLS to select subsets of correlated features (genes and cytokines) in an integrative framework.

HIV-LIPO-5 vaccine is a mix of five synthetic HIV-1 peptides (from Gag, Pol, Nef), each coupled to a palmytoil tail. PBMCs from 12 HIV-negative volunteers were collected before (W0) and after vaccination (W14). PBMC were stimulated with either i) the HIV-LIPO-5 vaccine; or ii) a pool of 15-mer Gag peptides included in the HIV-LIPO-5 vaccine (Gag+); or iii) a pool of 15-mer peptides not included in LIPO-5 vaccine (Gag-). Production of 10 cytokines was assessed at day 11 (MILLIPLEX MAP kit, Millipore). Gene transcription in PBMC was assessed after 6- and 24-hour stimulations (Illumina Human HT12-v4 chips).

## Results

After vaccination, the multilevel discriminant analysis led to the selection of 290 genes over three components that gave a good separation of the four groups of stimulation. The first component that distinguished LIPO5 stimulation from the others included a cluster of genes belonging to the metallothionein family (MT1M, MT2A,…) possibly linked to the effect of the palmytoil tail. The second component separated Gag+ from other stimulations. The multilevel sPLS showed similar profiles of correlations between gene expression and cytokine secretion for TH2 (IL5 and IL13), IL21 and IL1b, TNF and IL6.

## Conclusion

This new statistical approach helps in analyzing complex designs. In VAC18, it revealed the differential responses to vaccine peptides and the lipid adjuvant. Gene expression signatures associated with cytokine responses were identified.

